# Comparing the risk factors related to intra-familial and extra-familial sexual abuse in cases that underwent medicolegal evaluation and prepared a report

**DOI:** 10.1192/j.eurpsy.2025.1104

**Published:** 2025-08-26

**Authors:** F. Bayraktar, S. Aras

**Affiliations:** 1 Child and Adolescent Psychiatry, Dokuz Eylul University Faculty of Medicine, İzmir, Türkiye

## Abstract

**Introduction:**

Childhood sexual abuse, a serious issue for all societies, is a complex experience that requires many legal and professional initiatives(Odacı et al. Psikiyatride Güncel Yaklaşımlar,2023).It is a widespread, multidimensional problem worldwide, with physical, mental, social, moral, cultural, and legal aspects(İşeri et al. Çocuk ve Ergen Psikiyatrisi Temel Kitabı,2008).

**Objectives:**

This study aimed to evaluate risk factors and medicolegal assessment results in children who were sexually abused.

**Methods:**

The records of children who were victims of sexual abuse retrospectively reviewed at Dokuz Eylül University Faculty of Medicine, Department of Child and Adolescent Psychiatry, between January 2010 and December 2023.Sociodemographic, clinical, psychometric, and medicolegal evaluation data were analyzed.

**Results:**

Of the 537 cases that underwent medicolegal evaluation, 80.3% were male and 19.7% were female.The mean age at the time of sexual abuse incident was significantly higher in females(12.5±3.4) than in males(9.8±3.4).Before the incident, 63.0% of cases had at least one psychiatric diagnosis(most commonly Mental Retardation or Developmental Delay(57.5%), MDD(5%), and Conduct Disorder(4.7%)).The period after the incident, 77.1% had at least one psychiatric diagnosis(most commonly PTSD(57.9%) and MDD(49.7%)), most of which occurred after the incident.22.7% of the cases were victims of incest, 77.3% were victims of extra-familial abuse.The most common abuser was the father in incest(7.1%) and a familiar person(neighbor, etc.) in extra-familial abuse(22.4%).51.4% of cases experienced simple sexual abuse, while 48.6% faced sexual penetration.In 42.7% of the cases, sexual abuse was first disclosed to family members. In incest victims, compared to cases of extra-familial sexual abuse; the rates of being female(86.9%-78.3%), having successful-average academic success(55.2%-43.3%), having normal-borderline intelligence(77.7%-67.7%), having an unemployed father(25.5%-16.2%), being subjected to simple sexual abuse(63.1%-48.0%), being subjected to sexual abuse by a single perpetrator(90.2%-81.2%), being subjected to repeated sexual abuse(69.7%-45.8%), living in a broken family(66.7%-38.9%), not having a psychiatric diagnosis before the incident(50.8%-34.2%), and withdrawing the abuse allegation/complaint(18.9%-6%) were statistically significantly higher.The age at the date of the incident was significantly younger(median:11-13), and the time taken to apply to judicial authorities (months) was significantly longer(median:18.5-12).Predictive variables of incest have also been evaluated using logistic regression analysis.

**Conclusions:**

In this study, the findings found in cases that underwent medicolegal evaluation support the literature findings regarding risk factors for sexual abuse and the negative effects of sexual abuse on children and adolescents.

**Disclosure of Interest:**

None Declared

